# Transplanted hair follicle stem cells migrate to the penumbra and express neural markers in a rat model of cerebral ischaemia/reperfusion

**DOI:** 10.1186/s13287-020-01927-w

**Published:** 2020-09-23

**Authors:** Xuemei Zhang, Hao Tang, Senlin Mao, Bing Li, Yinglian Zhou, Hui Yue, Duo Wang, Yifei Wang, Jin Fu

**Affiliations:** Department of Neurology, The Second Affiliated Hospital of Harbin Medical University, No.246 Xuefu Road, Nangang District, Harbin, 150086 Heilongjiang Province China

**Keywords:** Hair follicle stem cells, Ischaemia/reperfusion, Cell transplantation, Homing, Differentiation

## Abstract

**Background:**

Ischaemic stroke has become the main cause of death and severe neurological disorders, for which effective restorative treatments are currently limited. While stem cell transplantation offers therapeutic potential through neural regeneration, this approach is associated with the challenges of limited applicable sources. Hair follicle stem cells (HFSCs) are multipotential cells that can differentiate into ectodermal and mesodermal lineages and proliferate for long periods. The therapeutic potentials of HFSCs have not been investigated in ischaemic stroke models, and therefore, in this study, we aimed to determine whether they could survive and migrate to ischaemic areas after a stroke attack.

**Methods:**

A rat model of middle cerebral artery ischaemia/reperfusion was established and intravenously administered HFSCs. The potential of HFSCs to migrate and differentiate into neuron-like cells as well as their ability to reduce the infarct size was evaluated. Rat brain tissue samples were collected 2 weeks after cell transplantation and analysed via TTC staining, immunofluorescence and immunohistochemistry methods. The data were statistically analysed and presented as the means ± standard deviations.

**Results:**

Intravenously administrated rat HFSCs were able to migrate to the penumbra where they expressed neuron-specific markers, reduced the infarct volume and promoted neurological recovery.

**Conclusion:**

HFSC transplantation has therapeutic potential for ischaemic stroke and is, therefore, worthy of further investigation toward possible clinical development for treating stroke patients.

## Background

Ischaemic stroke has become the main cause of disability and death worldwide [1]. The number of patients who can undergo recanalization therapy is restricted because of the strict eligibility criteria and narrow time window [2, 3]. The central nervous system (CNS) can hardly regenerate under pathological conditions, which leads to irreversible neurological disabilities. However, regenerative medicine brings new hope for functional organ reconstruction.

For many CNS diseases, such as spinal cord injury and stroke, stem cell transplantation is a practical regenerative strategy; however, it is associated with inevitable challenges. For example, neural or embryonic stem cells are suitable stem cell sources for CNS diseases; however, they are very sparse and extremely difficult to acquire. In contrast, bone marrow mesenchymal or hemopoietic stem cells, which have been used in many diseases, originate from a different embryo layer than the neural system, and thus, they have limited potential to differentiate into neurons [4, 5]. Moreover, the identified stem cell sources cannot meet the substantial clinical requirements in a short time.

Hair follicle stem cells (HFSCs) can be a highly promising source of multipotent cells, as they are abundant, accessible, and active in adult mammals. Throughout the lifetime, mammals’ hair undergoes a unique regenerative phenomenon consisting of cyclic periods, growth, regression, and rest. The stem cells contained in the hair follicle bulge region have been verified based on several criteria [6]. HFSCs continuously produce new cells to restore follicles and cutaneous appendages during the anagen phase [7] and can differentiate into most mesodermal and ectodermal derivatives [8]. Despite skin diseases, these characteristics make HFSCs a promising cell source for the treatment of nervous system disorders [9].

Stem cells isolated from the rat vibrissa have been shown to differentiate into ectodermal lineages such as neural cells, astrocytes, oligodendrocytes, and Schwann cells when cultured in vitro [10], as well as mesodermal derivatives including myocytes [11], chondrocytes [12], and osteocytes [13]. Interestingly, Robert M. Hoffman proved that after 7 days of culture in RPMI 1640 medium containing 10% FBS, 48 ± 8%, and 30 ± 8% of the population of HFSCs differentiated into neurons and glial cells, respectively [14]. Moreover, HFSCs have been transplanted into skin injury [15, 16], spinal cord injury [17], peripheral nerve injury [6], and Alzheimer’s disease animal models [18], with results indicating their potential to localise to the injury site and promote angiogenesis or neurological functional recovery.

As a promising source of stem cells for ischaemic stroke treatment, HFSCs have three important advantages making them prominent among all stem cells. First, they are abundant and accessible. Second, they are pluripotent, holding the capability to differentiate into both ectodermal and mesodermal lineages. Third, they are active and proliferative for long periods, making it possible for most patients to accept auto-transplantation without ethical or logistic problems. However, HFSCs have not been evaluated in ischaemic stroke models to date, and whether they can survive and migrate to ischaemic areas after a stroke attack is still unclear. Therefore, in the present study, we aimed to investigate this phenomenon using a middle cerebral artery (MCA) ischaemia/reperfusion (I/R) rat model based on immunohistochemical, immunofluorescence and TTC staining assays.

## Materials and methods

### Experimental design

A total of 48 rats were divided into four groups at random: (i) control, (ii) sham, (iii) I/R + saline and (iv) I/R + HFSCs. Rats in the control group were healthy and not subjected to an operation, whereas the I/R model was established via surgical MCA occlusion (MCAO). In the sham group, rats received a similar operation but without MCA occlusion. The I/R + HFSCs group underwent HFSC transplantation (1 × 10^6^ cells dispersed in 1 mL saline) via tail vein injection 24 h after reperfusion, whereas animals in the I/R + saline group and sham group were similarly administered 1 mL saline.

**Fig. 1:**
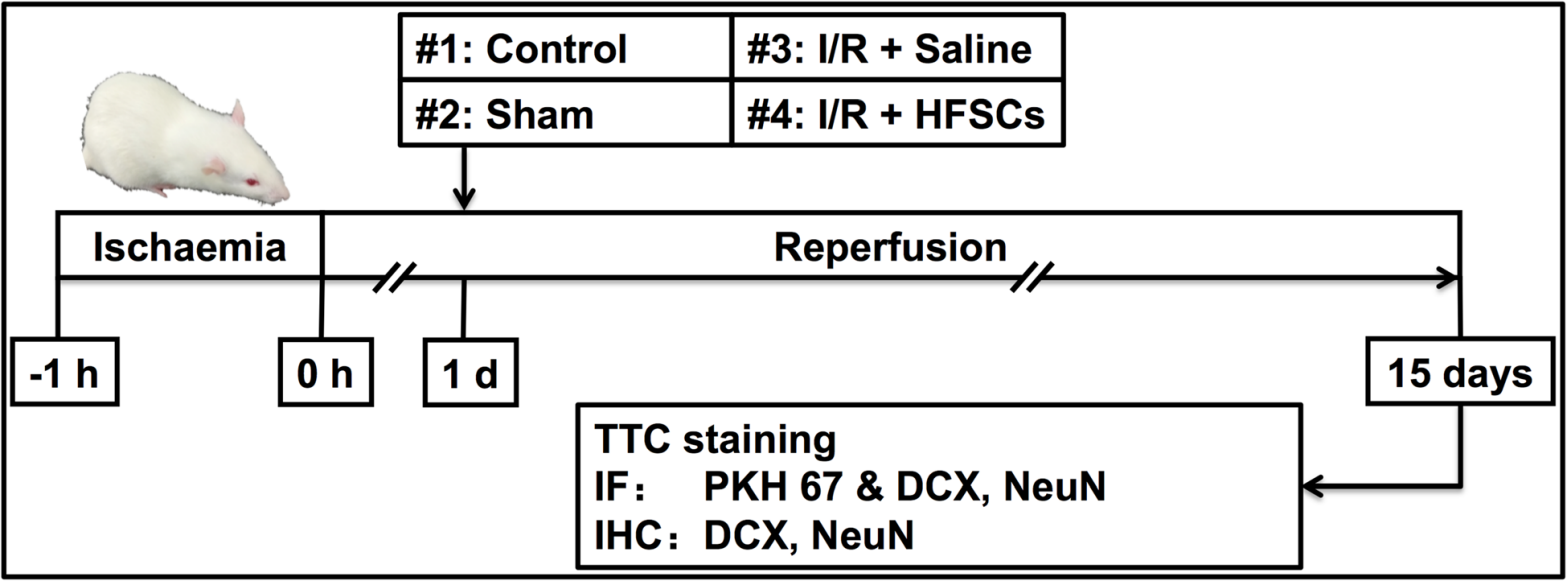
Experiment design and procedure. The rats in sham group and I/R + saline group were treated with 1 mL saline, whereas rats in I/R + HFSC group were administrated with HFSCs (1 × 10^6^ in 1 mL saline) transplantation, 24 h after reperfusion. Behavioural tests were conducted daily after reperfusion until 14 days post-transplantation. TTC staining, immunofluorescence, and immunohistochemistry assays were conducted on day 15

Animals were maintained for 2 weeks after transplantation, and then, relevant tissue samples from half of the animals were analysed using TTC (Amresco, OH, USA) and the other half were used for histological staining (*n* = 6 each, Fig. 1). The 2-week survival point was chosen because it might be the shortest time required for the transplanted cells to migrate to the penumbra and exert putative protective effects on stroke recovery. Neurological scores (Table 1) were recorded daily after cell transplantation [19]. The score of each rat was estimated three times. A full score represents a normal neurological status, whereas lower scores are indicative of behavioural deficits.

**Table 1:** Neurological scores system

	Score			
	0	1	2	3
Spontaneous activity (3-min test period)	No movement	Slight movement	Touches 1 or 2 sides of cage	Touches 3 or 4 sides of cage
Symmetry of movement (forelimbs and hind limbs)	Total asymmetry	Near-total asymmetry	Mild asymmetry	Complete symmetry
Floor walking	No walking	Walks in circles only	Curvilinear path	Straight path
Beam walking	Falls off of beam	Hugs beam	Stands on beam	Walk on beam
Response to vibrissae touch of the left side		No response	Weak response	Symmetrical response

### Animals

Male Sprague-Dawley (S-D) rats weighing 280 ± 10 g were purchased from the animal centre of the Second Affiliated Hospital of Harbin Medical University. The animals were housed at 22 ± 2 °C with a humidity of 40 ± 5%, under a 12-h light/dark cycle, and fed a standard diet and water ad libitum. The rats were forbidden to eat 12 h before the experiments but were allowed free access to drinking water. All study design and experimental procedures were conducted in accordance with institutional guides for animal experiments approved by the Experimental Center of the Second Affiliated Hospital of Harbin Medical University.

### Isolation, culture, labelling and transplantation of rat-derived HFSCs

We harvested hair follicles via enzyme digestion following mechanical dissection [20]. The upper lips containing the vibrissa pad of 4-week-old male S-D rats were cut and digested with 0.1% collagenase in DMEM (both from Gibco, BRL, Gaithersburg, MD, USA). Then, the vibrissa follicles were gently plucked from the pad under a binocular microscope, placed in 24-well tissue-culture dishes (Corning, NY, USA) pre-treated with IV collagen, and cultured in DMEM containing 10% FBS (ScienCell, Santiago, CA, USA) and 1% penicillin and streptomycin (Gibco, BRL, Gaithersburg, MD, USA) at 37 °C in an atmosphere of 95% air–5% CO_2_. All surgical procedures were conducted in accordance with aseptic principles, and the entire culture medium was first replaced with fresh medium 12 h later. The non-adherent cells were discarded with the waste culture medium, and thereafter, the culture medium was replaced every 48 h. When the culture grew to approximately 80% confluency (~ 10 days), the new adherent cells were passaged using the same culture method. The prepared HFSCs were used at passage 3 in the experiments. To monitor the grafted cells in the brain, the HFSCs were pre-labelled by non-invasive membrane-labelling green fluorescent dye PKH 67. Two weeks after HFSC transplantation, the TTC and histological staining were performed.

### Induction of focal cerebral I/R

The focal cerebral I/R model was induced by the right MCAO for 1 h following reperfusion, as reported previously [21, 22]. Briefly, after intraperitoneal anesthetisation with compound anaesthetic consisting of 4.25% chloral hydrate and 0.886% pentobarbital sodium (0.3 mL/100 g), a midline ventral incision was made on the neck to expose the vessels. The right external carotid artery (ECA) was isolated, and the branches were cauterised. The right MCA was occluded by gently inserting a monofilament nylon suture with a rounding tip through the right ECA. After a 60-min occlusion, the suture was slowly pulled back to achieve reperfusion. The rats in the sham group received the similar procedure except that the right MCA was not occluded. The body temperature was maintained at 37 ± 5 °C using a thermostat-controlled heating pad from the start of the operation until the animals recovered from anaesthesia.

### TTC staining

To measure the infarct volume, TTC staining was carried out as described previously [23]. Briefly, brain tissue was cut into five coronal slices (2-mm thick) and incubated in 1% TTC dissolved in PBS for 15 min at 37 °C. The non-infarcted tissue was stained red, whereas the infarcted tissue area remained white. The infarct volume was calculated as follows: [(left hemisphere area − right non-infarcted area)/(left hemisphere area × 2)] to avoid the influence of oedema [24].

### Nissl staining

Following anaesthesia, rats were transcardially perfused with 0.9% saline until no blood flowed out, followed by 4% paraformaldehyde in PBS (pH 7.4). The brain tissue was removed and kept in 4% paraformaldehyde for 48 h, cryoprotected in 30% sucrose in PBS for another 48 h at 4 °C and then embedded in OCT compound (Sakura Finetek, Torrance, CA, USA). The brain specimens were cut into coronal slices at a 10-μm thickness between the optic chiasma and the cerebral caudal end using a cryostat machine (Thermo Scientific Microm HM560, Waltham, MA, USA). The sections were then air-dried and processed for pathological experiments, including Nissl staining. Nissl bodies were stained using Cresyl violet acetate (Sigma-Aldrich, St Louis, MO, USA). Briefly, the brain tissue slices were immersed in the Cresyl violet acetate solution for 2 h at 37 °C, successively dehydrated and hyalinised, and observed under an optical microscope [21].

### Immunofluorescence

Frozen sections were blocked with 5% goat serum (Cat. No. abs933, Absin, Shanghai, China) in PBS for 30 min and incubated with primary rabbit anti-doublecortin (Cat. No. ab18723, DCX, Abcam, Cambridge, UK), mouse anti-neuron-specific nuclear protein (Cat. No. MAB377, NeuN, Millipore Corp, Billerica, MA, USA) antibodies overnight at 4 °C. After rinsing with PBS, the sections were incubated with rhodamine-conjugated anti-rabbit IgG (1:500, Cat. No. 4413S, Cell Signalling Technology, VT, USA) or anti-mouse IgG (1:500, Cat. No. 4409S, Cell Signalling Technology, VT, USA) for 90 min at 25 °C. The nuclei were stained with DAPI. PKH 67 (green), neuron-specific markers (red) and DAPI (blue) were observed using laser scanning confocal microscopy (Zeiss LSM800; Carl Zeiss, Jena, Germany) at wavelengths of 594 nm (red), 488 nm (green) and 405 nm (blue), respectively.

### Immunohistochemistry

The brain sections were incubated with 0.3% H_2_O_2_ to eliminate endogenous peroxidase, blocked with 10% goat serum and treated with 0.1% triton X-100 in PBS for 30 min at 25 °C. Then, the sections were incubated with rabbit anti-DCX, mouse anti-NeuN antibodies overnight at 4 °C. After washing with PBS, the sections were incubated with horseradish peroxidase-linked anti-rabbit or anti-mouse IgG (1:500, Cell Signalling Technology, VT, USA) for 60 min at 25 °C and stained with DAB (Cell Signalling Technology, VT, USA). The sections were rinsed with PBS, counterstained with haematoxylin, then dehydrated and observed using an optical microscope.

### Statistical analysis

The statistical analysis was performed using GraphPad Prism 6.0 (GraphPad Prism Software, San Diego, CA, USA). The data were analysed using one-way ANOVA, followed by Tukey’s test for multiple comparisons and Student’s *t* test for two group comparisons. Values with *P* < 0.05 were considered statistically significant. All data are expressed as means ± standard deviations (SDs).

## Results

### Characteristics of HFSCs

**Fig. 2:**
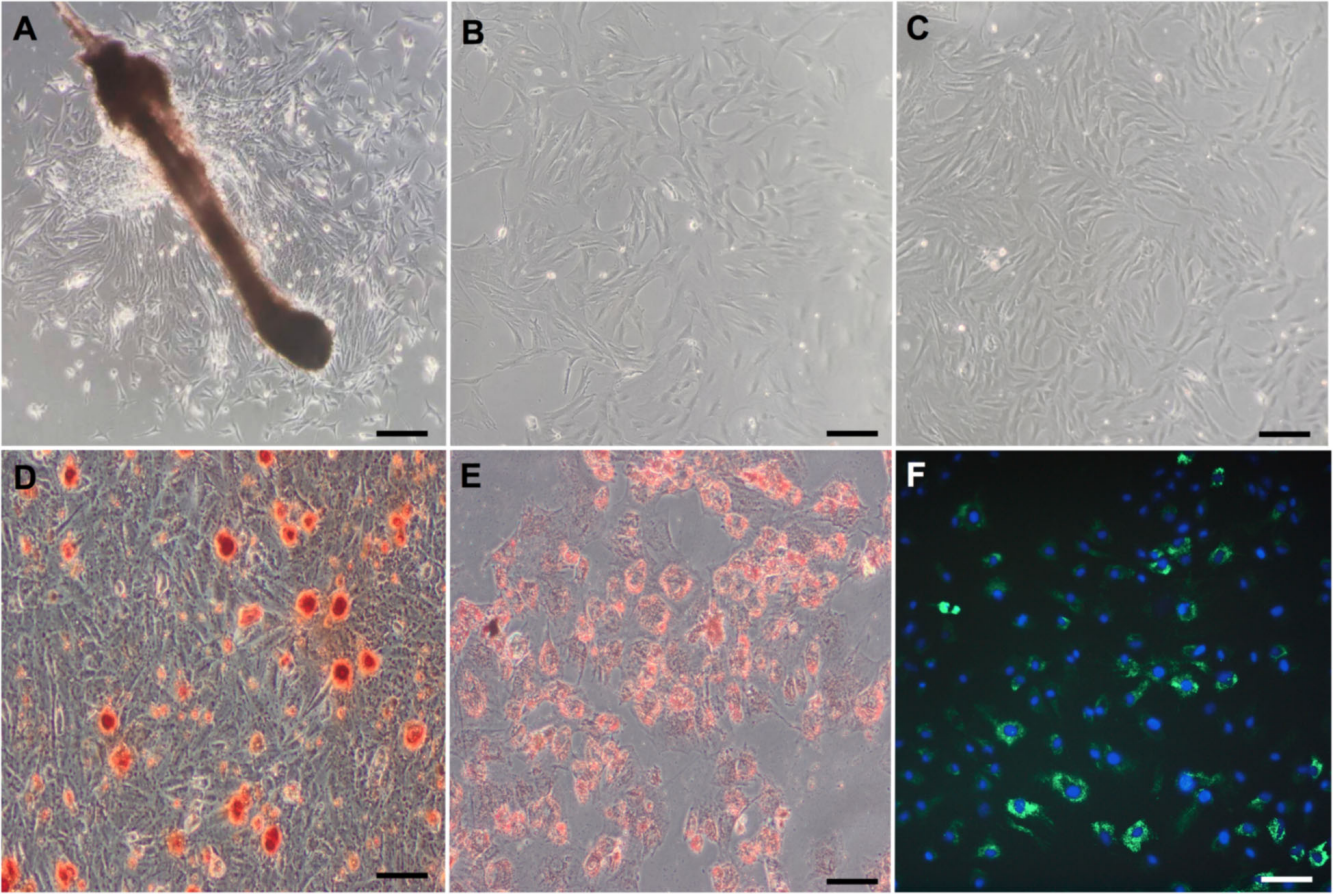
Identification of hair follicle stem cells. **a** P0: Primary HFSCs started to form colonies 7–10 days and attached to the plate surface. **b**, **c** P1 and P3: HFSCs displayed triangular and paving stone-like morphology. **d**, **e** Multilineage differentiation of HFSC: mineralised nodules and fat droplets. **f** PKH67-labelled HFSCs emitted green fluorescence under fluorescence microscope. The nuclei were stained blue with DAPI. Scale bar = 50 μm

**Fig. 3:**
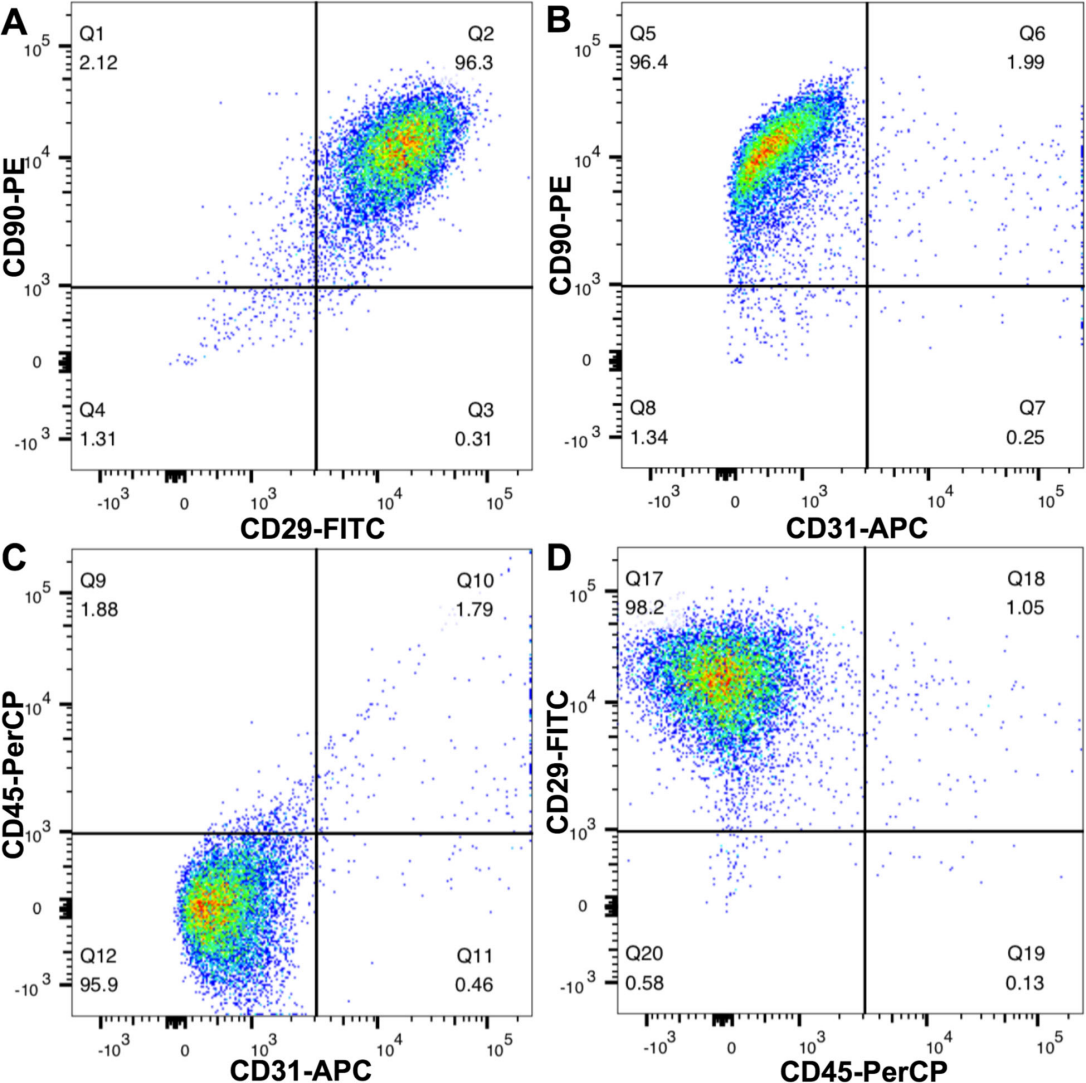
Fluorescence-activated cell sorting (FACS) of HFSCs. CD29 and CD90 are surface markers of mesenchymal stem cells. CD31and CD45 are the surface antigens of endothelial cells and leucocytes, respectively. The cultured HFSCs mainly expressed CD29 and CD90, rarely expressed CD31and CD45

The self-renewable HFSCs exhibited colony formation, plastic adherence and a paving stone-like morphology (Fig. 2a–c). The capacity of multipotent differentiation was verified by osteogenesis and adipogenesis experiments (Fig. 2d, e). The image of PKH-67-pre-labelled HFSCs under a fluorescence microscope is presented in Fig. 2f. The expression of mesenchymal stem cell surface markers was detected by fluorescence-activated cell sorting (FACS) (BD FACS Canto II, NJ, USA) analyses (Fig. 3). In conclusion, the cells derived from S-D rat hair follicle vibrissa appeared to be largely HFSCs.

### Focal cerebral I/R rat model

**Fig. 4:**
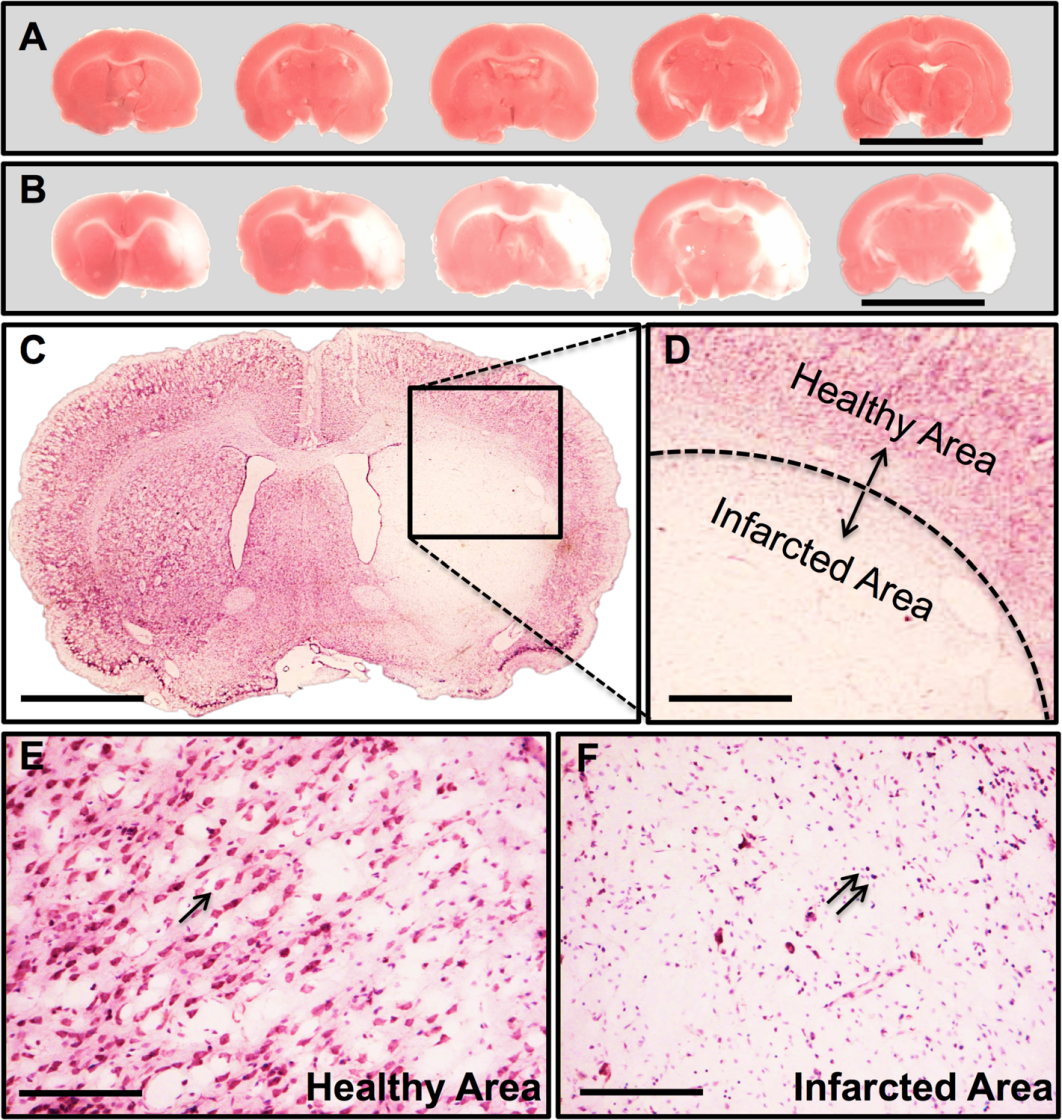
MCAO modelling assessment. **a** Consecutive coronal slices of sham operated rats. Scale bar = 20 mm. **b** Serial coronal slices of MCAO rats. Typical photographs of rat brain stained with 2,3,5-triphenyltetrazolium chloride (TTC), wherein no infarction tissue was stained red, whereas the infarction area remained white. Scale bar = 20 mm. **c**, **d** Nissl staining of MCAO models revealed lesions in the brain tissues, with diminished numbers of neurons and chaotic neuronal configuration. Scale bar = 5 mm (**c**) and 1.5 mm (**d**). **e**, **f** Enlargement of healthy area and infarcted area. The black arrow indicates Nissl-positive neurons. The double arrows indicate nuclear pyknosis with karyorrhexis. Scale bar = 100 μm

TTC staining and Nissl staining were used to confirm and analyse the successful establishment of the focal cerebral I/R model along with neurological system scores. A white zone within the brain tissue slices represented the infarcted area caused by I/R injury, whereas the red zone represented normal brain tissue (Fig. 4a, b). Nissl staining (Fig. 4c, d) showed significant damage to neurons in the infarcted area. Neurons in the healthy area exhibited normal morphological features, whereas those in the infarcted area showed several impaired features such as cell loss, nuclear karyorrhexis, and pyknosis.

### HFSCs migrate to ischaemic penumbra

**Fig. 5:**
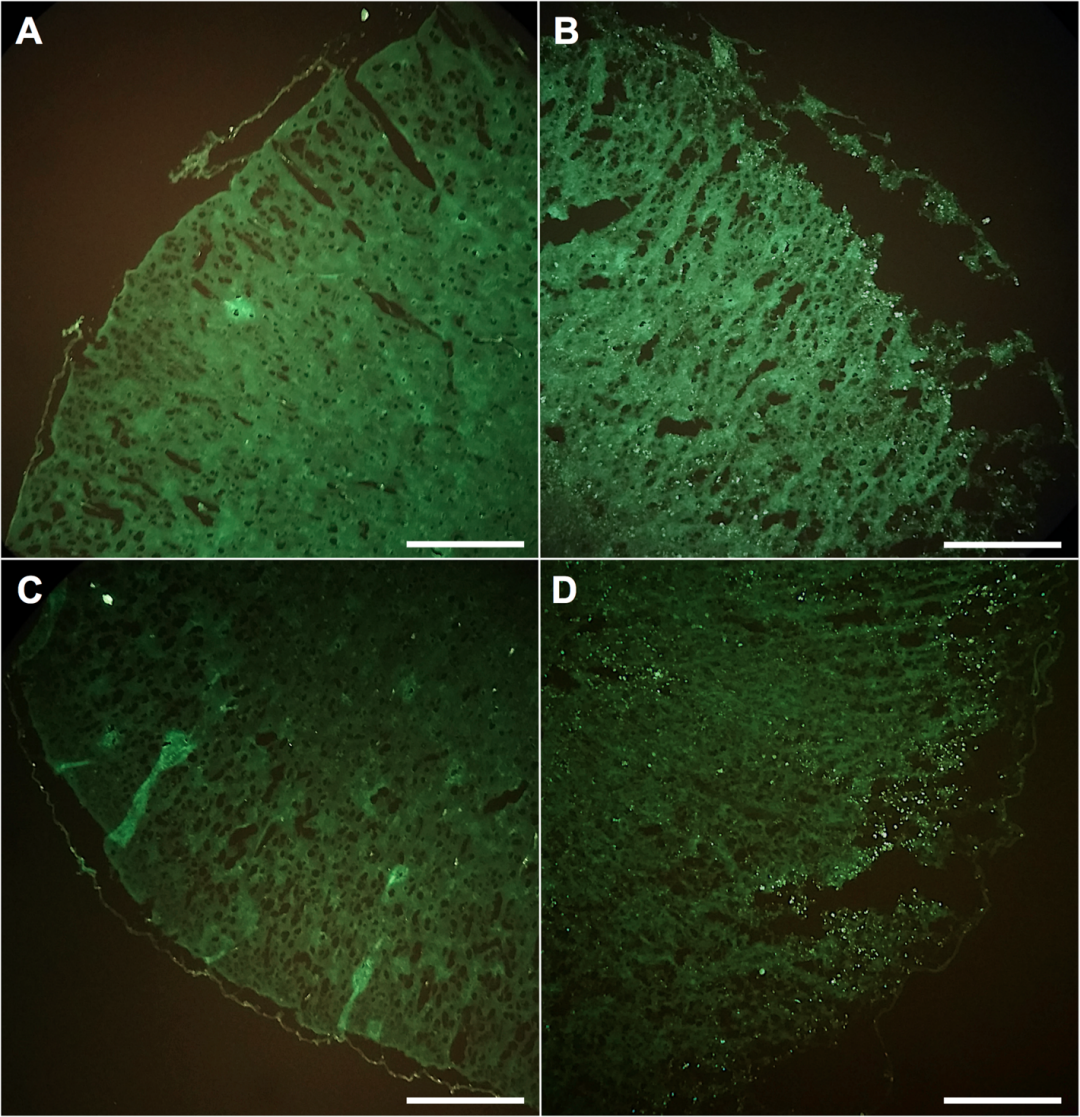
PKH67-labelled hair follicle stem cells (HFSCs) present in the penumbra. **a**, **c** Different regions of contralateral hemisphere of I/R. **b**, **d** Typical regions of the hemisphere with I/R injury. Majority of PKH67-labelled HFSCs gathered around the ischemic area, but rarely migrated to the contralateral hemisphere. Scale bar = 1 mm

The spatial distribution of HFSCs within the brain was monitored and analysed after transplantation into the cerebral I/R model. The green fluorescent dye-pre-labelled HFSCs were easily identified using a fluorescent microscope (Fig. 5). Figure 5a, c shows the regions of the non-infarcted hemisphere, whereas images of the I/R-insulted tissue are presented in Fig. 5b, d. PKH 67-labelled HFSCs were visibly localised to the penumbra area, but rarely found in the normal hemisphere.

### Grafted HFSCs express neuron-specific markers

**Fig. 6:**
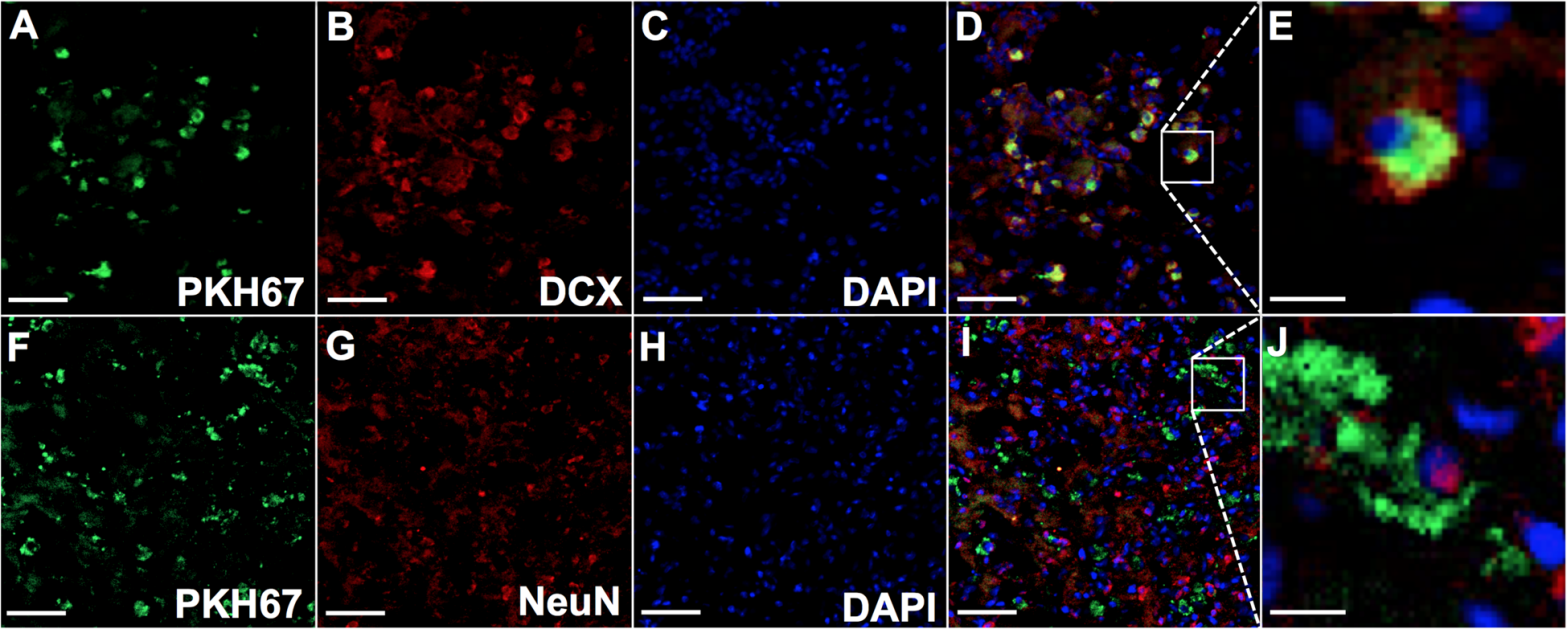
HFSCs in the penumbra express neuron-specific markers including doublecortin (DCX) and neuron-specific nuclear protein (NeuN). **a**, **f** PKH-67-labelled HFSCs emitted green fluorescence in the penumbra. **b**, **g** The neuron-specific markers with Alexa Fluor® 594-conjugated secondary antibodies emitted red fluorescence. **c**, **h** DAPI staining emitted blue fluorescence at the wavelength of 405 nm. **d**, **i** PKH67-labelled HFSCs were overlayed with the panel of neuron-specific markers and DAPI. Scale bar = 50 μm. **e**, **j** The magnified typical image of co-localised cells. Scale bar = 10 μm

The co-localisation of PKH 67-pre-labelled HFSCs and neuron-specific markers was evaluated by laser scanning confocal microscopy. Cell tracker PKH 67 emitted green fluorescent signals both in vitro and in vivo. Immunolabelling of neural-specific markers conjugated with red fluorescent indicator was used to detect the differentiation activity of HFSCs in the I/R model. As shown in Fig. 6, PKH 67-labelled cells located at the penumbra region could express the neuron-specific markers DCX and NeuN, whereas DAPI stained the nuclei blue.

### Neuron-specific marker expression in penumbra

**Fig. 7:**
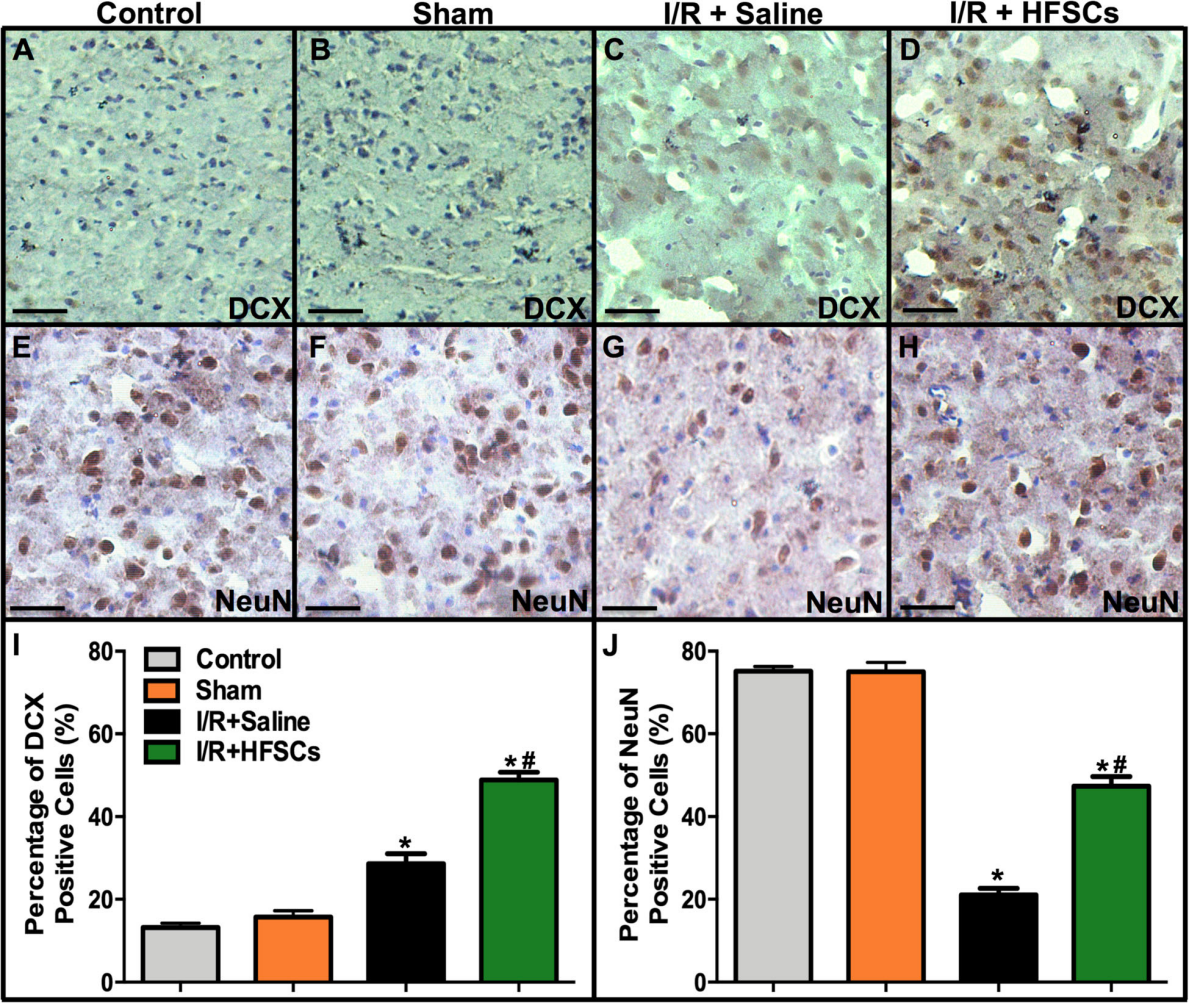
The expression of neuron-specific markers DCX and NeuN in the penumbra. **a–h** The representative photographs of neuron-specific markers expression in all groups. Scale bar = 25 μm. **i, j** The quantitation of the neuron specific markers positive cells. The number of positive cells was upregulated in HFSC group compared with I/R + saline group by immunohistochemistry assay. Values are the mean ± SD. *P < 0.05 vs. sham group, ^#^*P* < 0.05 vs. I/R group, (*n* = 6)

Immunohistochemistry staining was conducted to analyse the number of neurons in the penumbra regions. Figure 7 shows the number of neuron-specific marker-positive cells around the ischaemic core. The number of DCX-positive cells increased in the I/R + saline group compared with the sham group, while HFSC treatment enhanced the expression of DCX after I/R injury (*P* < 0.05). The number of NeuN-positive cells decreased in the I/R + saline group compared with that in the sham group, whereas these decreases were significantly inhibited by HFSC treatment (*P* < 0.05).

### HFSCs reduce infarct volume and improve neurological scores

**Fig. 8:**
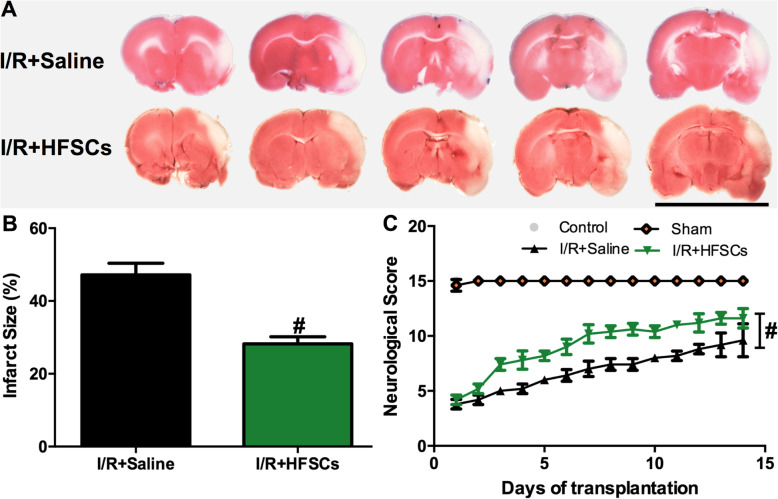
The effect of HFSCs on brain infarction and neurological scores. **a** Representative photographs of rat brain stained with TTC in different groups. Scale bar = 20 mm. **b** Quantitative analysis of infarct size in all groups, showing that infarct volume in saline group is about 44.20 ± 2.16%, whereas the HFSC transplantation group is to 29.80 ± 1.86%. **c** Neurological scores of animals in different groups. Values are the mean ± SD. *P < 0.05 vs. sham group, ^#^*P* < 0.05 vs. I/R group, (*n* = 6)

To investigate whether intravenously transplanted HFSCs could alleviate damage in the cerebral I/R model, TTC staining was conducted to measure the infarct volume 2 weeks after cell transplantation (Fig. 8). The TTC staining photographs of the I/R groups are exhibited in Fig. 8a, where the red and white colours indicate non-infarcted and infarcted tissue, respectively. Compared to that in the I/R + saline group, the I/R + HFSCs group showed a significant reduction in I/R-induced cerebral infarction.

Quantitation of the cerebral infarct volume showed that the proportion was 44.20 ± 2.16% in the I/R + saline group, whereas in the I/R + HFSCs group, the white zone was significantly smaller at 29.80 ± 1.86% (*P* < 0.05) (Fig. 8b). The neurological score indicated that the rats in the I/R + saline group underwent slow recovery from neurological deficits induced by I/R injury, whereas HFSC transplantation obviously improved neurological functional recovery (*P* < 0.05) (Fig. 8c).

## Discussion

In this study, we demonstrated that transvenously grafted rat HFSCs could survive in a cerebral I/R rat and migrate to the ischaemic boundary zone. Moreover, most PKH67-labelled HFSCs expressed the neuron-specific cell markers DCX and NeuN in the penumbra area. Furthermore, HFSC injection exerted a protective effect on I/R-induced brain injury by reducing the infarct volume and enhancing neurological functional recovery. These phenomena suggested that HFSCs could be a novel alternative source for ischaemic stroke regenerative treatment with remarkably appealing potential.

Stem cell transplantation, as a promising therapeutic approach in stroke diseases, has been investigated for many years in numerous studies [25]. However, obtaining an optimal cell source has been challenging. HFSCs are considered an outstanding candidate among different stem cells because of their attractive characteristics such as abundance, easy accessibility, low invasiveness, multipotency, and auto-transplantability with no ethical or logistical problems.

Since HFSCs have not been investigated in a cerebral I/R rat model, we prudently considered the administration route and transplantation time window. Firstly, intravenous infusion, compared with other administration routes such as intraarterial or intraventricular routes, is more suitable for the administration of stem cells, with the least invasive injury or risk of thrombosis. In the present study intravenous administration proved to be a safe and effective approach and could be a practical method for the clinical application of these findings. Secondly, an optimal time point for transplanted stem cell survival after a stroke exists before the maximal activation of the microglia [26]. In a stroke rat model, the corresponding time window would be within 2–3 days after the insult, which is when the maximum accumulation of macrophages is observed [27]. For this reason, we chose to conduct HFSC transplantation 24 h after reperfusion. Thirdly, the HFSCs differentiated into neural class III β-tubulin-positive and CD31-negative neurons 2 weeks after transplantation into nude mice [14]. Thus, in the present study, the brain tissue was collected 2 weeks after cell transplantation. DCX, a valuable marker of immature neuron, has been used to detect neurogenesis [28]. NeuN is usually used to mark mature neurons [29, 30]. Our study demonstrated that 2 weeks after transplantation into the cerebral I/R rat model, HFSCs migrated to the ischaemic penumbra and expressed neuron-specific cell markers (Fig. 6), as expected.

We first verified the homing capability of HFSCs in a cerebral I/R rat model after intravenous administration. Studies have reported that homing mechanisms of stem cells, including HFSCs, to sites of ischaemia requires a coordinated multistep process, including recruitment of stem cells, adhesion to the endothelium, transendothelial migration, matrix degradation and invasion, and in situ differentiation [31–33]. Stromal cell-derived factor 1 (SDF-1) and its receptor C-X-C motif chemokine receptor 4 (CXCR4) play vital roles as the drivers of stem cells homing [34, 35]. A recent study suggests that platelets participate in metastasis and homing of stem cells by spatial approach and direct contact, which indicates a new main source of homing-related factors [36, 37]. In the present study, we focused on elucidating the cell homing phenomenon by performing pathological experiments. The cytokines and factors involved in HFSC homing after transplantation into I/R rats thus require further study.

Transplanted mesenchymal stem cells possess the potential to differentiate into neurons [38]. The results showed that the grafted HFSCs in the penumbra expressed neuron-specific markers, thereby indicating that they might differentiate into neuron-like cells such as immature neurons expressing DCX or mature neuron expressing NeuN (Fig. 6). Stem cell differentiation might partially account for the increase in neuron-specific marker-positive cells in the HFSC group (Fig. 7). Additionally, many studies have reported that stem cells contribute to neuroprotection via pro-angiogenesis [39, 40], pro-neurogenesis [41], anti-inflammation and anti-apoptosis [42] mechanisms in I/R injury, which could be concluded to represent paracrine effects. Recently, co-culture studies in vitro using a transwell set-up have revealed that mesenchymal stem cells alleviate acute lung injury via paracrine effect of hepatocyte growth factor [43]. Moreover, FGF-2-modified human gingival mesenchymal stem cells have been verified to promote angiogenesis via paracrine effects of angiogenesis-related growth factors [44]. Besides, HFSCs have been demonstrated to possess neuroendocrinology [45] and ability to promote recovery of peripheral nerve injury by paracrine effects [46]. Thus, combined with the results of the present study, the neural protection effects induced by HFSCs might be partially mediated via their paracrine ability, but further studies are needed to confirm this hypothesis. What can be verified from our study is that HFSC transplantation reduced neuron loss and the infarct volume and improved neurological recovery (Fig. 8).

## Conclusions

In conclusion, our results illustrate a homing phenomenon in which transvenously grafted HFSCs localised around the ischaemic regions, without requiring a blood–brain barrier permeabilizer, and expressed neuron-specific cell markers. Moreover, HFSCs exhibited therapeutic effects by reducing the infarct volume and promoting neurological functional recovery, which require more fundamental studies to illuminate the underlying mechanisms before application in a clinical setting.

## Data Availability

The datasets used and/or analysed during the current study are available from the corresponding author on reasonable request.

## Abbreviations

HFSCs: Hair follicle stem cells
I/R: Ischaemia/reperfusion
MCAO: Middle cerebral artery occlusion
