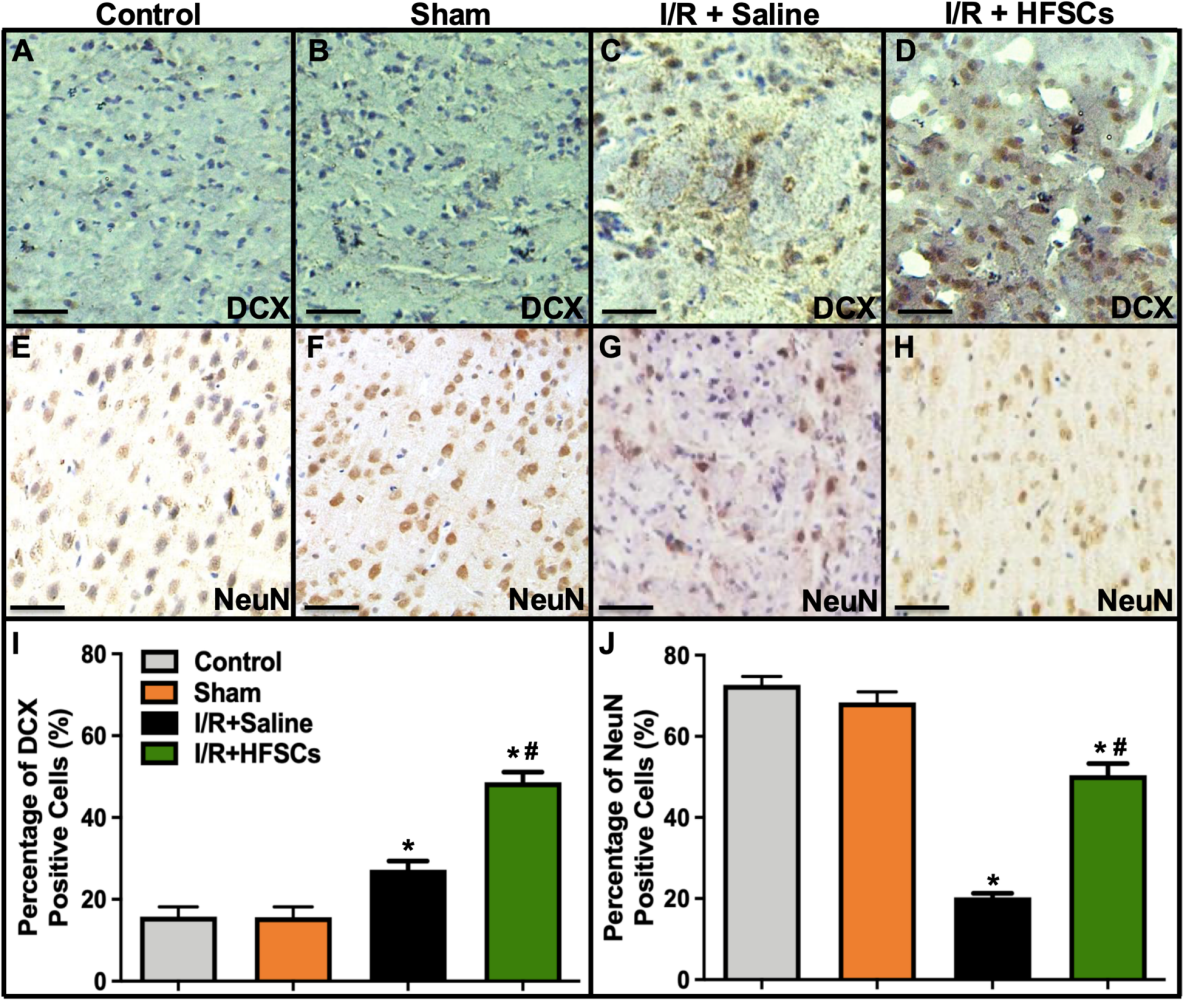# Correction: Transplanted hair follicle stem cells migrate to the penumbra and express neural markers in a rat model of cerebral ischaemia/reperfusion

**DOI:** 10.1186/s13287-025-04864-8

**Published:** 2025-12-20

**Authors:** Xuemei Zhang, Hao Tang, Senlin Mao, Bing Li, Yinglian Zhou, Hui Yue, Duo Wang, Yifei Wang, Jin Fu

**Affiliations:** Department of Neurology, The Second Affiliated Hospital of Harbin Medical University, No.246 Xuefu Road, Nangang District, Harbin, 150086 Heilongjiang Province China

**Correction: Stem Cell Research & Therapy (2020) 11:413**

10.1186/s13287-020-01927-w

During an internal review, the authors identified an inadvertent misplacement of immunohistochemistry (IHC) images due to a renaming error during the image-saving process. Specifically, in the NeuN staining group, microscope images of brain slices from 1 rat were mistakenly assigned to different groups (Fig. 7E–H), and in the DCX staining group, one image (Fig. 7D) was also misfiled. Unfortunately, several of the misfiled images were selected as representative figures due to personal visual preference, and the issue was not detected during figure preparation.

The authors deeply regret this oversight and have carefully re-verified the original data. The affected images have now been replaced with the correct ones, and the revised Fig. 7 can be viewed ahead in this Correction article. Additionally, the authors reassessed the statistical analysis without misfiled images and confirmed that the reported trends and significance remain unchanged. The authors confirm that: this correction does not affect the overall conclusions of the study; the key finding that "HFSC transplantation could migrate to the penumbra to alleviate ischemia–reperfusion injury" is supported by multiple lines of evidence, including immunofluorescence assays, IHC assays, TTC staining, and behavioral experiments; and that the integrity of the findings remains intact.